# Can the Prognostic Nutritional Index Be a Predictor of Acute Exacerbation of Idiopathic Pulmonary Fibrosis?

**DOI:** 10.3390/biomedicines14092088

**Published:** 2026-09-16

**Authors:** Mustafa Çolak, Hikmet Çoban

**Affiliations:** Department of Pulmonology, Balıkesir University, 10145 Balıkesir, Türkiye; hikmet.coban@balikesir.edu.tr

**Keywords:** idiopathic pulmonary fibrosis, acute exacerbation, prognostic nutritional index

## Abstract

**Background/Objectives:** Idiopathic pulmonary fibrosis (IPF) is a fibrotic lung disease that can be accompanied by acute exacerbations. To investigate the predictive value of the prognostic nutritional index (PNI) for acute exacerbation in patients with IPF. **Methods:** Ninety-seven patients diagnosed with IPF between 03/2019 and 03/2024 were retrospectively evaluated. Patients were divided into two groups according to exacerbation status (acute exacerbation group *n* = 28, non-acute exacerbation group *n* = 69). PNI values were calculated. The predictive value of the PNI for acute exacerbation was analyzed between groups. ROC analysis and multivariable logistic regression were performed. **Results:** Of the 97 patients, 78 (80.4%) were male with a mean age of 71.31 ± 6.61 years. The mean disease duration was 24.77 ± 15.69 months. During follow-up, 28 patients (28.9%) had acute exacerbations. Lymphocyte count and albumin level were significantly lower in the acute exacerbation group (*p* < 0.001, *p* < 0.001, respectively). The mean PNI value was significantly lower in the acute exacerbation group (46.62 ± 6.28) compared to the non-acute exacerbation group (52.04 ± 5.46) (*p* < 0.001). In addition, FEV_1_%, FVC%, and DLCO% values were significantly lower in the acute exacerbation group (*p* < 0.001, *p* < 0.001, *p* < 0.001, respectively). In ROC analysis, the cut-off value of PNI ≤ 48.50 had a sensitivity of 64.29% and specificity of 78.26% (AUC:0.744, *p* < 0.001). In multivariable logistic regression analysis, the PNI (OR = 0.857, *p* = 0.007) and FVC% (OR = 0.949, *p* = 0.008) were independent predictors of acute exacerbation. **Conclusions:** The PNI may serve as a negative biomarker for predicting acute exacerbation risk in IPF patients. This simple, cost-effective parameter could be useful in risk assessment.

## 1. Introduction

Idiopathic Pulmonary Fibrosis (IPF) is a disease of unknown cause that leads to progressive fibrotic lung damage and decreases survival [1]. Despite advances in anti-fibrotic therapies, the median survival time of IPF ranges between 2.5 and 4 years [2,3]. Acute exacerbation characterized by sudden clinical deterioration is the most lethal complication of IPF [4]. In a meta-analysis published in 2024, 11.855 IPF patients were evaluated and the annual incidence of exacerbation was reported as 9% and the 3-year incidence as 19% [5].

Understanding the mechanisms driving this progressive course requires consideration of the underlying pathogenesis of IPF, which results from an interplay of several interconnected processes. Inflammatory and immune dysregulatory mechanisms have been implicated in fibroblast activation and excessive extracellular matrix deposition in IPF; however, the extent to which inflammation drives disease progression remains debated, given the central role of fibroblast dysfunction in fibrogenesis [6,7].

Beyond inflammatory and immune mechanisms, oxidative stress—arising from an imbalance between reactive oxygen species and antioxidant defenses—contributes to epithelial cell injury and fibrogenesis. Network pharmacology analyses have also implicated oxidative stress-related targets and NF-κB signaling in pulmonary fibrosis [8,9]. Endothelial injury and vascular remodeling may further disturb pulmonary tissue homeostasis and contribute to fibrogenesis through dysregulated profibrotic signaling [10]. Finally, impaired repair following recurrent epithelial injury, marked by aberrant wound healing and persistent myofibroblast activation, is considered a central driver of progressive fibrosis; recent evidence implicates molecular regulators such as the PARP1-FOXN3 axis in controlling this process [11,12].

Since acute exacerbation of IPF (AE-IPF) has a high mortality rate, early diagnosis and determination of the risk level are of great importance. Various prognostic models have been developed for this purpose. Nutritional status is one of the factors affecting prognosis and quality of life in IPF patients. In a multicenter study, it was revealed that patients diagnosed with IPF may have malnutrition in the early period [13]. Malnutrition in IPF is associated with increased respiratory muscle load, inflammatory activity, coexisting hypoxemia, and reduced physical activity, which may further impair clinical status and adversely affect clinical outcomes [14]. Awano et al. showed that low body mass index (BMI) was associated with poor prognosis in patients with AE-IPF [15]. Serum albumin levels have also been defined as one of the predictors of survival in IPF patients [16].

The prognostic nutritional index (PNI) is a parameter calculated by serum albumin level and total lymphocyte count [PNI = albumin (g/L) + total lymphocyte count (10^9^/L) × 5] [17]. Although the PNI was initially developed for preoperative nutritional status assessment, there have been studies showing its prognostic value in various cancers in recent years [18,19]. In a meta-analysis published by Li et al., low PNI was reported to be an independent prognostic factor in lung cancer patients [20]. Chen et al. emphasized the role of immune mechanisms in acute exacerbations in IPF and stated the importance of parameters reflecting immunity [21]. More broadly, acute exacerbation involves an abrupt intensification of several pathobiological processes implicated in IPF, including dysregulated inflammation, immune imbalance, oxidative stress, and alveolar epithelial cell apoptosis [22]. As the PNI integrates nutritional and immune status—two clinically relevant dimensions of systemic vulnerability in IPF—it may represent a plausible biomarker for predicting acute exacerbation risk.

In the current literature, data on the prediction of the prognostic nutritional index in IPF patients, especially acute exacerbation, are limited. In our study, we aimed to investigate the value of the prognostic nutritional index in predicting the development of acute exacerbation in IPF patients.

## 2. Materials and Methods

Adult patients (aged ≥ 18 years) followed up with a diagnosis of IPF in the Chest Diseases Clinic of our hospital between 03/2019 and 03/2024 were included in our study. IPF diagnosis was established according to the current ATS/ERS/JRS/ALAT 2022 guidelines [2] and through multidisciplinary team discussion. Exclusion criteria were as follows: history of active cancer, active infection period, history of hematologic disease, history of rheumatologic disease, hepatic and/or renal failure, and use of immunosuppressive therapy. Patients with these conditions were excluded from the study, considering the potential effects on nutritional status and hematological parameters. Additionally, patients whose initial presentation was acute exacerbation were also excluded from the study. Demographic data, body mass index, comorbidities, Charlson comorbidity index (CCI), disease duration, anti-fibrotic treatments, and history of IPF acute exacerbation were evaluated in outpatient clinic evaluations. Complete blood count, albumin, c-reactive protein (CRP), sedimentation, and prognostic nutritional index (PNI) values, as well as pulmonary function tests, diffusion tests, dyspnea scoring (mMRC), and GAP (Gender–Age–Physiology) indices were retrospectively analyzed. All laboratory parameters, including serum albumin and lymphocyte count used for the PNI calculation, were obtained at the time of diagnosis during a clinically stable period, prior to any acute exacerbation event. In the acute exacerbation group, a sufficient interval was observed between the baseline PNI measurement and the occurrence of acute exacerbation, confirming that the PNI reflected a stable-phase assessment rather than a response to the exacerbation itself. Two groups were formed according to the exacerbation history of the patients. IPF acute exacerbation cases were identified and adjudicated according to the international consensus criteria defined by Collard et al. (2016) [4], which require an acute, clinically significant respiratory deterioration characterized by evidence of newly developing diffuse alveolar abnormality on high-resolution computed tomography, occurring within 30 days, without an identifiable alternative cause. Alternative etiologies, including pneumonia, heart failure, and pulmonary embolism, were systematically excluded based on clinical evaluation; further diagnostic workup—including microbiological cultures, echocardiography, natriuretic peptide measurement, or CT pulmonary angiography—was performed when clinically indicated. All suspected acute exacerbation events were reviewed and confirmed through multidisciplinary team discussion. The primary aim was to evaluate whether the PNI could serve as a predictor of future acute exacerbation in patients with IPF. The follow-up period for each patient was defined as the interval from the date of PNI measurement at diagnosis to either the occurrence of acute exacerbation, last clinic visit, or the end of the study period (March 2024). The observation time therefore varied among patients depending on their date of enrollment. Cases with missing laboratory or functional data required for PNI or GAP calculation were excluded from the analysis on a variable-wise basis, and no imputation method was applied for missing data. Written informed consent was obtained from all participants prior to their inclusion in the study.

## 3. Statistical Analysis

All analyses were performed with IBM SPSS Statistics 23.0. The power analysis performed using G*Power (version 3.1.9.7)indicated that a total sample size of 97 patients would provide 95% statistical power to detect a clinically significant difference in the primary outcome at a two-sided significance level of α = 0.05 (Cohen’s d = 0.95). The estimated power for secondary outcomes was above 80%. Continuous variables were summarized as mean ± standard deviation, categorical variables as number (*n*) and percentage (%). The Kolmogorov–Smirnov test was used for normal distribution between groups, and chi-square tests (Pearson’s χ^2^ and Fisher’s exact) were used for categorical variables. For normally distributed continuous data, the difference between the two groups was analyzed using the Independent Samples *t*-test, and Welch’s *t*-test was used when the assumption of variance homogeneity was not met. The Mann–Whitney U test was used for non-normally distributed data. Pearson’s correlation coefficient (r) was used for linear relationships between variables, and Spearman’s rank correlation coefficient (ρ) was used for non-normally distributed or ordinal data. The discriminatory power of the binary outcome measure was assessed by ROC analysis. The effect of multiple independent variables on the binary dependent variable was analyzed by logistic regression analysis, and odds ratios and 95% confidence intervals were presented. A multivariable Cox proportional hazards regression analysis was also performed to account for differences in observation time, with hazard ratios (HR) and 95% confidence intervals reported. Additionally, a separate logistic regression model including the PNI and the GAP index was performed to assess the incremental predictive value of the PNI. In all analyses, *p* < 0.05 was considered significant.

This study was conducted in accordance with the principles of the Declaration of Helsinki and was approved by the Health Research Ethics Committee of Balıkesir University (approval number 2025-3/10, dated 24 June 2025).

## 4. Results

The study included 97 patients with IPF. The mean age of the patients was 71.31 ± 6.61 years. Seventy-eight patients (80.4%) were male. The mean disease duration of all patients was 24.77 ± 15.69 months. The majority of patients (81 patients, 83.5%) had at least one comorbidity. When the use of anti-fibrotic treatment was analyzed, 49 patients were using nintedanib and 44 patients were using pirfenidone. Four patients were followed up without treatment. Demographic and clinical characteristics of the patients are presented in Table 1.

Patients were divided into two groups in terms of acute exacerbation history: 28 patients with acute exacerbation and 69 patients without acute exacerbation. There was no significant difference between the groups in terms of mean age (*p* = 0.29). The groups showed similar characteristics in terms of gender distribution (*p* = 0.78) (Table 2).

When the mean body mass index (BMI) was evaluated, no significant difference was found between the groups (*p* = 0.97). Charlson comorbidity index (CCI) median values were 4.75 in the non-acute exacerbation group and 4.50 in the acute exacerbation group, and there was no statistically significant difference between the groups (*p* = 0.55) (Table 2).

The median disease duration was comparable between the two groups, with no statistically significant difference observed (*p* = 0.72). Since none of the patients in the non-AE group experienced an acute exacerbation, their disease duration values also represent their total follow-up time. The median duration from the time of diagnosis to acute exacerbation occurrence was 9.71 (2–63) months in the exacerbation group. There was no significant difference between the groups in terms of the anti-fibrotic treatment agent used (*p* = 0.53). Two patients in each group were followed up without treatment (Table 2).

When complete blood count parameters were analyzed, leukocyte, hemoglobin, neutrophil, and platelet values were similar between the groups (*p* = 0.50, 0.79, *p* = 0.20, *p* = 0.45, respectively). Lymphocyte median values were significantly lower in the acute exacerbation group (*p* < 0.001) (Table 2).

Although C-reactive protein and sedimentation median values were higher in the IPF acute exacerbation group compared to the non-acute exacerbation group, there was no statistically significant difference between them (*p* = 0.08, *p* = 0.37, respectively).

Albumin median values were significantly higher in patients in the non-acute exacerbation group than in patients with exacerbation (*p* < 0.001). The mean prognostic nutritional index was 52.04 ± 5.46 in the non-acute exacerbation group and 46.62 ± 6.28 in the exacerbation group, and a statistically significant difference was found between the groups (*p* < 0.001) (Table 2).

Pulmonary function tests indicated significant functional impairment in the group with acute exacerbation. The mean values of forced vital capacity (FVC%) and 1. second forced expiratory volume (FEV_1_ %) were significantly lower in the acute exacerbation group (*p* < 0.001, *p* < 0.001, respectively) (Table 2).

Diffusion capacity (DLCO%) was significantly lower in the acute exacerbation group compared to the non-acute exacerbation group (*p* < 0.001). GAP (Gender–Age–Physiology) index median values were significantly higher in the acute exacerbation group compared to the non-acute exacerbation group (*p* < 0.001) (Table 2).

The relationship between the PNI and clinical and functional parameters was analyzed. No significant correlation was found between the PNI and disease duration, Charlson comorbidity index, and body mass index (*p* = 0.50, *p* = 0.14, *p* = 0.12, respectively) (Table 3).

In terms of inflammatory markers, a weak negative correlation was found between the PNI and C-reactive protein (*p* < 0.001) (Table 3).

Correlations between pulmonary function tests and the PNI were analyzed. No significant correlation was found between the PNI and FEV_1_% and FVC% (*p* = 0.19, *p* = 0.23, respectively).

The strongest correlation supporting the prognostic value of the PNI was found between the PNI and diffusion capacity (DLCO%), positively and moderately (*p* < 0.001). A weak negative correlation was observed between the PNI and GAP index (*p* < 0.001) (Table 3).

**Figure 1:**
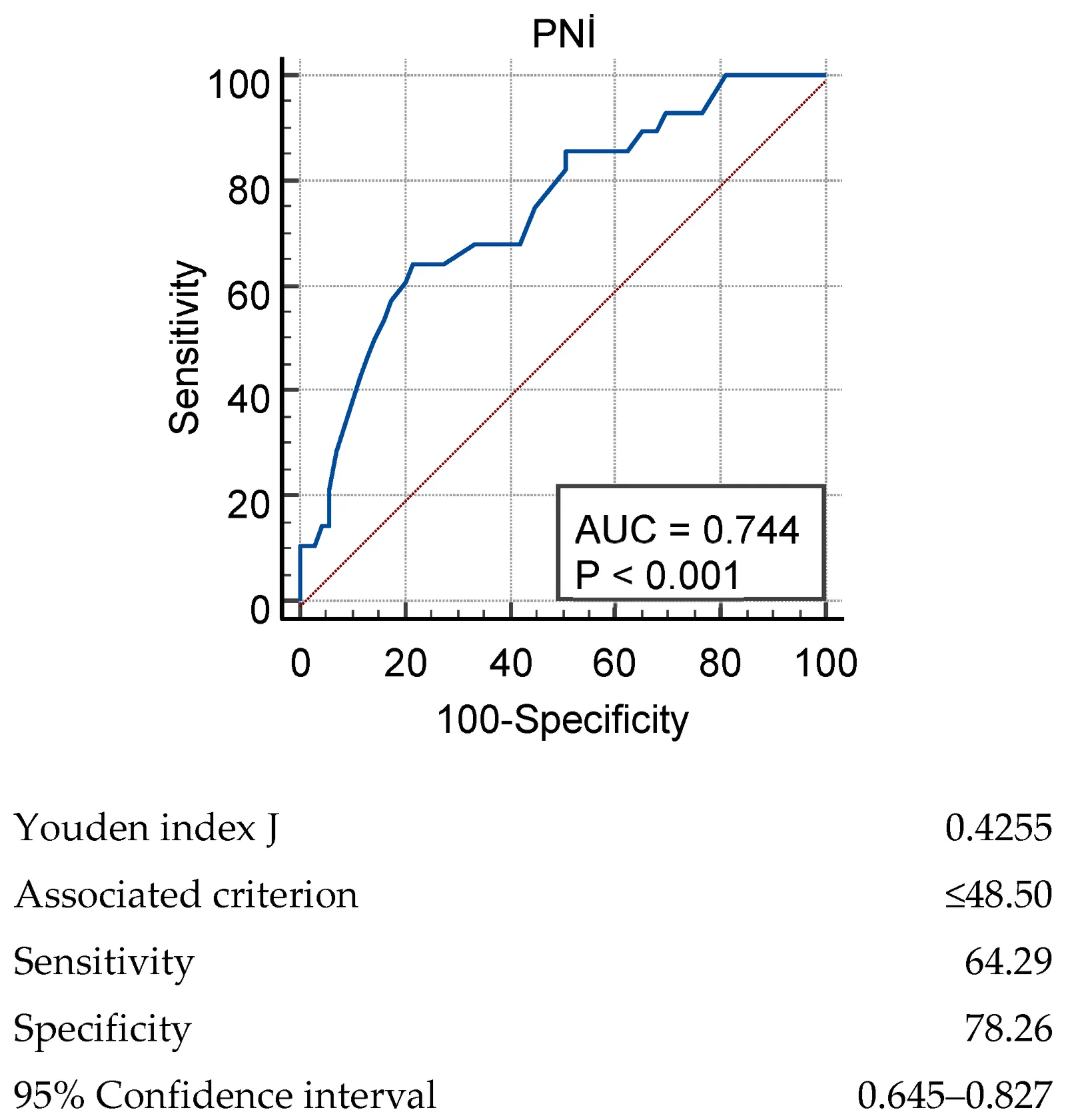
ROC analysis results for PNI in assessing acute exacerbation of IPF.

ROC analysis for the prediction of IPF acute exacerbation by the PNI score yielded an AUC of 0.744 (*p* < 0.001). Youden J was used to evaluate the predictive power of the PNI for IPF acute exacerbation. For a cut-off value of PNI ≤ 48.50, *p* = 0.001, sensitivity was 64.29% and specificity was 78.26% (Figure 1).

Retrospective multivariable logistic regression analysis was performed to identify independent predictors of acute exacerbation in IPF. Variables were selected based on statistical significance in univariate analyses. The GAP index was excluded due to its collinearity with FVC% and DLCO%, and serum albumin and lymphocyte count were not entered separately as they are the constituent components of the PNI. The PNI and FVC% were found to be independent predictors of acute exacerbation. Each unit increase in the PNI reduced the risk of exacerbation by 14.3% (OR = 0.857, 95% CI: 0.765–0.959, *p* = 0.007), while each unit increase in FVC% reduced the risk of exacerbation by 5.1% (OR = 0.949, 95% CI: 0.912–0.986, *p* = 0.008). In contrast, DLCO% had no statistically significant effect on acute exacerbation (*p* = 0.061) (Table 4). The overall fit of the model was good (Omnibus Test: χ^2^(4) = 34.169, *p* < 0.001), and the Hosmer–Lemeshow test showed that the model fit the data (χ^2^(8) = 5.146, *p* = 0.742). The model correctly classified exacerbation status 77.8% of the time (Nagelkerke R square = 0.439).

To further evaluate whether the PNI provides predictive value beyond an established composite severity measure, a separate multivariable logistic regression model including the PNI and the GAP index was performed. The PNI remained an independent predictor of acute exacerbation even after adjusting for the GAP index (OR = 0.864, 95% CI: 0.781–0.955, *p* = 0.004), while the GAP index itself was also independently associated with exacerbation risk (OR = 2.491, 95% CI: 1.461–4.246, *p* = 0.001). The model showed good fit (Hosmer–Lemeshow test: χ^2^ = 2.743, *p* = 0.949; Nagelkerke R^2^ = 0.402).

A multivariable Cox proportional hazards regression analysis was additionally performed to account for differences in observation time, using the same covariates as the logistic regression model (PNI, FVC%, and DLCO%). Consistent with the logistic regression findings, the PNI remained an independent predictor of acute exacerbation, with each unit decrease increasing the risk by 6.5% (HR = 0.935, 95% CI: 0.886–0.987, *p* = 0.015). FVC% was also confirmed as an independent predictor, with each unit decrease increasing the risk by 3.3% (HR = 0.967, 95% CI: 0.942–0.992, *p* = 0.011). In contrast to the logistic regression model, DLCO% reached statistical significance in the Cox model, with each unit decrease increasing the risk by 3.2% (HR = 0.968, 95% CI: 0.938–0.999, *p* = 0.043).(Table 5). The overall model was statistically significant (Omnibus Test: χ^2^(3) = 31.699, *p* < 0.001).

## 5. Discussion

In our study, we observed that the prognostic nutritional index (PNI) may be a significant negative biomarker for predicting acute exacerbation of IPF. Acute exacerbation was significantly higher in patients with PNI ≤ 48.50. In multivariable logistic regression analysis, The PNI was identified as an independent predictor of acute exacerbation. The PNI was positively correlated with DLCO%.

The demographic characteristics of the patients in our study were consistent with the literature. The male predominance of 80.4% in our cohort overlaps with the literature reporting that IPF is more common in males. IPF is a disease diagnosed at an advanced age [1]. In our study, the mean age of our patients was 71.31 ± 6.61 years, which is compatible with the literature data. In the real life study by Isshiki et al., the 3-year incidence of IPF-AE was found to be 20.4–29.6% in patients receiving treatment, depending on the anti-fibrotic agent used [23]. In a recent study, the 5-year incidence of acute exacerbation in patients with pulmonary fibrosis was found to be 38% [24]. In our study, the rate of acute exacerbation was 28.9%, which was in a range compatible with the literature.

The significantly lower lymphocyte count in the acute exacerbation group emphasizes the role of the immune system. Suzuki et al. showed that higher lymphocyte levels in blood or bronchoalveolar lavage fluid were associated with a better prognosis in cases of interstitial lung disease [25]. The inclusion of lymphocytes in the calculation of the PNI in our study is important in terms of including immune system changes and being compatible with the literature. The significantly lower albumin levels in the acute exacerbation group are consistent with previous studies. Zisman et al. showed that serum albumin levels were an important marker in predicting survival in patients with idiopathic interstitial pneumonia [16]. Recent studies show that low albumin levels may be a risk factor for mortality in the 3-year follow-up of IPF patients [1].

The lower PNI values in the acute exacerbation group emphasize the importance of nutritional status on the prognosis of IPF. Jouneau et al. showed that malnutrition may be present in the early period in IPF patients, which is consistent with these findings [13]. As an index incorporating both nutritional status and immune function, the PNI has been investigated as a potential marker for predicting acute exacerbation and prognosis in various lung diseases. Suzuki et al. reported that the PNI was a potential predictor in COPD exacerbation [26]. In non-small cell lung cancer patients, the pre-treatment PNI has been shown to have prognostic value [27]. The cut-off value of PNI ≤ 48.50 determined by ROC analysis showed clinical significance in the prediction of acute exacerbation. This finding suggests that the PNI may help identify IPF patients at higher risk of exacerbation. The increasing interest in nutritional assessment and intervention in IPF supports the clinical significance of our findings [28,29].

In our current study, when CRP levels were compared between IPF patients evaluated during the diagnostic process and those in the stable clinical phase, no significant difference was found between the patient groups. This finding suggests that CRP may have limited value as a biomarker in the stable phase of IPF. Indeed, a study by Yang et al. showed that CRP levels were significantly higher in cases experiencing acute exacerbation compared to stable IPF patients [30]. This result indicates that CRP reflects the inflammatory burden, particularly during the acute exacerbation period, and that it may be expected to remain low or within normal limits during the stable phase. The fact that CRP measurements in our study were performed during the stable phase explains the lack of a significant difference between the groups and is consistent with the inflammation dynamics reported in the literature. Therefore, it can be concluded that while CRP is useful in identifying periods of acute exacerbation, it is a limited indicator for predicting exacerbation risk during the stable phase.

In our study, impairment in pulmonary function tests performed at the time of diagnosis (during the stable period) was an expected finding in the group experiencing acute exacerbation of IPF. A significant decrease in FVC% and especially DLCO% may indicate disease progression and the risk of acute exacerbation. Recent studies emphasize the prognostic importance of DLCO and FVC in IPF [31,32]. In our study, the finding that each unit increase in FVC% reduced the risk of exacerbation by 5.1% demonstrates the importance of preserving lung function. Although DLCO% did not reach statistical significance as an independent predictor in the logistic regression model, it emerged as a significant predictor in the Cox proportional hazards model, suggesting that its prognostic value may become more apparent when the time-dependent nature of exacerbation risk is taken into account.

The moderate correlation we found between the PNI and DLCO suggests that nutritional status may be related to lung function. This finding is consistent with the literature data suggesting that malnutrition may negatively affect respiratory muscle functions [33] and may accelerate the progression of the disease.

A significantly higher GAP index in the acute exacerbation group indicates an advanced stage of the disease. The GAP index is a prognostic tool consisting of age, gender, and physiologic parameters [34]. Although the GAP index was excluded from the primary multivariable model due to collinearity with FVC% and DLCO%, a supplementary analysis performed to assess the PNI’s incremental value beyond this composite severity score showed that the PNI retained independent prognostic significance even after adjustment for the GAP index. This suggests that nutritional status may have an additional contribution to disease prognosis.

## 6. Limitations

The main limitations of our study are its retrospective design, single center, and limited number of patients. In addition, the effect of nutritional interventions on the PNI and the effect of these interventions on the risk of acute exacerbation could not be evaluated. Due to these limitations, prospective and multicenter studies are needed to investigate the effectiveness of nutritional interventions.

## 7. Conclusions

Our study shows that the prognostic nutritional index may be a valuable biomarker for predicting the risk of acute exacerbation in IPF patients. The PNI is an easily applicable parameter that is simply calculable and cost-effective. We suggest that a cut-off value of PNI ≤ 48.50 could be considered for risk stratification in clinical practice. However, external validation of this proposed cut-off value in independent cohorts is needed before its routine clinical implementation. Our findings emphasize that nutritional status should be assessed in patients with IPF, especially at initial presentation and during follow-up, as systematic assessment may help identify those at higher risk of exacerbation. Whether nutritional intervention can modify this risk warrants further investigation in prospective, multicenter studies.

## Figures and Tables

**Table 1:** Demographic and clinical characteristics of IPF patients.

Variable	*n* (%)
Age	
mean ± sd	71.31 ± 6.61
Gender	
Male	78 (80.4)
Female	19 (19.6)
Comorbidity	
Yes	81 (83.5)
No	16 (16.5)
Acute exacerbation	
Yes	28 (28.9)
No	69 (71.1)
Anti-fibrotic treatment	
Pirfenidon	44 (45.4)
Nintedanib	49 (50.5)
Untreated	4 (4.1)
Disease duration (month)	
mean ± sd	24.77 ± 15.69
mMRC score	
mean ± sd	1.84 ± 0.94

mMRC: Modified Medical Research Council.

**Table 2:** Comparison of patients with acute exacerbations and stable periods.

Variable	Non-AE Group (*n* = 69)	AE Group (*n* = 28)	*p*
Age			0.29
mean ± sd	70.86 ± 6.65	72.43 ± 6.48	
Gender			0.78
Female	14 (%20.3)	5 (%17.9)	
Male	55 (%79.7)	23 (%82.1)	
BMI			0.97
mean ± sd	27.97 ± 4.27	28 ± 4.12	
CCI			0.55
median (min–max)	4.75 (3–9)	4.50 (3–8)	
Anti-fibrotic treatment			0.53
Pirfenidon	33 (%47.8)	11 (%39.3)	
Nintedanib	34 (%49.3)	15 (%53.6)	
Untreated	2 (%2.9)	2 (%7.1)	
Disease duration			0.72
median (min–max)	22.67 (5–80)	22.86 (4–64)	
Leukocyte/µL	8.18 (4–15.5)	7.3 (4.7–13)	0.50
Hemoglobin g/L	13.92 (6.6–16.3)	13.63 (10.3–16.3)	0.79
Neutrophil/µL	4.86 (2.4–11.5)	5.05 (2.3–9.3)	0.20
Lymphocyte/µL	2.18 ± 0.67	1.71 ± 0.68	<0.001
Platelet/µL	264.14 ± 70.91	252.86 ± 58.05	0.45
Crp mg/L			0.08
median (min–max)	3.34 (1.1–63)	6.15 (3–53)	
Sedimentation			0.37
median (min–max)	21 (3–79)	29 (7–87)	
Albumin g/L			<0.001
median (min–max)	41.77 (30–48)	39.43 (25–44)	
PNI			<0.001
mean ± sd	52.04 ± 5.46	46.62 ± 6.28	
FEV_1_ %			<0.001
mean ± sd	88.03 ± 17.65	73.35 ± 16.53	
FVC %			<0.001
mean ± sd	82.83 ± 16.51	67.67 ± 14.82	
DLCO%			<0.001
mean ± sd	62.41 ± 20.20	46.29 ± 12.52	
GAP index			<0.001
median (min–max)	3.38 (1–6)	4.62 (3–6)	

BMI: Body mass index, CCI: Charlson comorbidity index, Crp: C-reactive protein, PNI: prognostic nutritional index, GAP: Gender–age–physiology index.

**Table 3:** The relationship between PNI and clinical and functional parameters.

Variable	r	*p*
Disease duration	0.069 (ρ)	0.50
BMI	0.158 (r)	0.12
CCI	−0.148 (ρ)	0.14
Crp	−0.326 (ρ)	<0.001
Sedimentation	−0.027 (ρ)	0.80
FEV_1_%	0.135 (r)	0.19
FVC%	0.123 (r)	0.23
DLCO%	0.408 (ρ)	<0.001
GAP index	−0.348 (ρ)	<0.001

ρ: Spearman Rank Correlation Coefficient, r: Pearson Correlation Coefficient.

**Table 4:** Results of multivariable logistic regression analysis.

Variable	B	Wald χ^2^	*p*	Exp(B)	%95 CI for OR	
					Lower	Upper
PNI	−0.155	7.164	0.007	0.857	0.765–0.959	
FVC%	−0.053	7.004	0.008	0.949	0.912–0.986	
DLCO%	−0.038	3.517	0.061	0.962	0.924–1.002	

Model summary: Nagelkerke R square:0.43, Hosmer–Lemeshow Test Chi-Square:5.146 *p*: 0.742.

**Table 5:** Results of Cox regression analysis.

Variable	B	Wald χ^2^	*p*	Exp(B)	%95 CI for OR	
					Lower	Upper
PNI	−0.067	5.944	0.015	0.935	0.886–0.987	
FVC%	−0.034	6.418	0.011	0.967	0.942–0.992	
DLCO%	−0.033	4.105	0.043	0.968	0.938–0.999	

Model Summary: −2 Log Likelihood: 192.849, Omnibus Test Chi-Square: 31.699, df: 3, *p* < 0.001.

## Data Availability

The datasets analyzed during the current study are available from the corresponding author on reasonable request.
